# Plant biomacromolecule delivery methods in the 21st century

**DOI:** 10.3389/fgeed.2022.1011934

**Published:** 2022-10-14

**Authors:** Sachin Rustgi, Salman Naveed, Jonathan Windham, Huan Zhang, Gözde S. Demirer

**Affiliations:** ^1^ Department of Plant and Environmental Sciences, School of Health Research, Clemson University Pee Dee Research and Education Center, Florence, SC, United States; ^2^ School of Agriculture and Biology, Shanghai Jiao Tong University, Shanghai, China; ^3^ Department of Chemical Engineering, California Institute of Technology, Pasadena, CA, United States

**Keywords:** biomacromolecule delivery methods, genome editing, CRISPR, RNA interference, genetic transformation, site-directed mutagenesis, nanoparticles, plants

## Abstract

The 21st century witnessed a boom in plant genomics and gene characterization studies through RNA interference and site-directed mutagenesis. Specifically, the last 15 years marked a rapid increase in discovering and implementing different genome editing techniques. Methods to deliver gene editing reagents have also attempted to keep pace with the discovery and implementation of gene editing tools in plants. As a result, various transient/stable, quick/lengthy, expensive (requiring specialized equipment)/inexpensive, and versatile/specific (species, developmental stage, or tissue) methods were developed. A brief account of these methods with emphasis on recent developments is provided in this review article. Additionally, the strengths and limitations of each method are listed to allow the reader to select the most appropriate method for their specific studies. Finally, a perspective for future developments and needs in this research area is presented.

## Introduction

There has been an exponential increase in the availability of genomic information for plant species in the last two decades, from the complete genomic sequence of *Arabidopsis thaliana* (The Arabidopsis Genome Initiative, 2000) to the pangenomes of maize (Hufford et al., 2021) and wheat (Walkowiak et al., 2020). However, the understanding of gene function has not caught up with the pace of gene discovery. The main reasons for this hindrance are the ability to transform only a limited number of plant species (and only a few selected genotypes within a species) and the time and effort required to transform a selected genotype. This lag between the mounting genomic information and gene characterization further highlights the need to develop or improve upon existing biomolecule delivery methods and, if possible, eliminate the need for trans-differentiation, tissue culture, and genotype-dependence. The US National Science Foundation acknowledged this need with the creation of the Plant Transformation Challenge Grants (TRANSFORM-PGR) under the Plant Genome Research Program (PGRP) in 2016.

This review article covers the established and newer biomacromolecule delivery methods and recently investigated variations of conventional methods. Substantial progress has also been made on the use of morphogenic genes (developmental regulators) to avoid genotype-dependence and tissue culture via inducing trans-differentiation of explants, supernumerary, or quiescent meristems and by promoting clonal propagation via seeds (parthenogenesis) by converting meiosis to mitosis (MeMi) and altering genes that induce haploidy. These topics, however, are out of the scope of this article, and readers interested in them are referred to the reviews by Khanday and Sundaresan, (2021), Kuo et al. (2021), and Chan, (2010).

The macromolecular cargo delivery methods discussed in this review article could be classified in various ways. For instance, they could be divided into conventional and novel methods depending on their usage. Furthermore, based on the mode of delivery, these methods could be classified into either direct or vector (biological/physical)-mediated cargo delivery methods. Based on the mode of delivery, these gene delivery methods could also be grouped under physical, chemical, or biological methods. Lastly, these biomacromolecule delivery methods could be classified as either transient/permanent or non-integrative/integrative. For ease of presentation, different delivery methods are discussed below based on a hybrid classification system that emphasizes both the conventionality of a method and mode of delivery.

## Conventional biomacromolecule delivery methods

In this section that focuses on conventional biomacromolecule delivery methods, the intent is not to provide a comprehensive historical account of their discoveries, as memoirs are available from the discoverers and can be consulted by interested readers (Paszkowski et al., 1984; Sanford, 2000; Chilton, 2001; Klein, 2011; van Montagu, 2011). Furthermore, this section will not document all research done using these conventional gene delivery methods, as there are specific books dedicated to these topics (Wang, 2015; Rustgi and Luo 2020). Instead, an effort is made to list certain modifications/updates to these procedures.

### Biolistic approach and its modifications

The discovery of biolistic (a portmanteau of “biological” and “ballistics”) biomolecule delivery was one of the finest agricultural innovations of the 20th century. It was one of two gene delivery methods available to researchers to genetically modify plants (Rustgi and Luo, 2020), the other being *Agrobacterium*-mediated gene delivery. *Agrobacterium*-mediated gene delivery slightly predated the biolistic method and has its limitations, such as genotype dependence, as it relies heavily on the gene interactions among the host genome, the *Agrobacterium* genome, and the Ti (tumor-inducer) or Ri (hairy root-inducing) plasmids (Christou, 1994, 1996). On the contrary, the major advantage offered by the biolistic method is its genotype independence, as it relies on physical force rather than biological interactions for biomolecule delivery. Some other advantages include the delivery of large DNA fragments (even some the size of whole bacterial artificial chromosome), which could be linear or circular (Yuan et al., 2012). The possibility of delivering linear DNA offers another advantage by eliminating the integration of the plasmid backbone (Yuan et al., 2012). Further, this method allows for a vast scope of alterations, in particular delivery of proteins, nucleoprotein complexes, and fluorescent dyes (technique dubbed ‘DiOlistics’) (Sherazee and Alvarez, 2013; Sudowe and Reske-Kunz, 2013; Rustgi and Luo, 2020). Other alterations involve research on the microprojectile (particle) size, type, and distance between the explant and the microprojectile accelerator or nozzle (Sanford, 2000). Some research also went into the development of the Hepta^™^ adaptor, which branches the acceleration tube in seven sections over seven macrocarriers to widen the field of particle delivery. Hence, it is supposed to uniformly spread the DNA-coated particles over a larger area and maximize the number of cells transformed during one bombardment. The Hepta^™^ adaptor for the PDS-1000/He biolistic system was claimed to have transformed 7–10 times more cells than the standard adaptor (https://www.bio-rad.com/). Since the gas is partitioned into seven sections, pressure and particle velocities are reduced, making it an ideal system for explant cultures and cell cultures requiring less forceful penetration. Research on microprojectiles and coating methods is ongoing (Ismagul et al., 2018; Cunningham et al., 2020). Recently, coating gold microcarriers with polyethylene glycol (PEG) and magnesium salt solutions, instead of spermidine and calcium chloride, substantially improved transformation frequency in common wheat (Ismagul et al., 2018).

The biolistic method remains one of the most used gene delivery methods in plants and was the source of most commercially released transgenics developed in the 1990s (Christou, 1996). The method is still evolving and has been modified to deliver nano-sized particles coated with nucleic acids/nucleoprotein complexes; a procedure termed “nanobiolistics” (O'Brien and Lummis, 2011; for detailed examination of this topic, see Cunningham et al., 2018, 2020). Another modification known as “Agrolistic transformation” combined the benefits of *Agrobacterium*-mediated gene delivery with the biolistic method. In this approach, the *Agrobacterium* virulence genes, virD1 and virD2 which are needed to liberate T-strands from the Ti plasmid in bacteria, were placed under the control of a constitutive cauliflower mosaic virus (CaMV) *35S* promoter and co-delivered via the biolistic approach with a target plasmid containing border sequences flanking the gene of interest. Hence, this approach offers the genotype independence of the biolistic method combined with the single-copy gene integration benefit of the *Agrobacterium*-mediated transformation method (Hansen and Chilton, 1996). This method was specifically used to modify difficult-to-transform crops, such as cotton and soybean (for details and later modifications of the method, see Basso et al., 2020).

Other modifications to the biolistic method include delivering proteins to plant and animal cells. This method, named “proteolistics,” requires the deposition of proteins along with microcarriers onto the macrocarrier surface. This does not require protein precipitation onto the micro-projectile surface, it does not involve any chemical modification of the microcarriers, and there is no chemical interaction between the protein and the microcarriers; hence, this method is not limited by the protein that can be delivered (Martin-Ortigosa and Wang, 2014, 2020). Despite the given advantages, this method did not gain much traction at the time of discovery until recently with the increased demand to deliver genome-editing reagents, i.e., guide RNAs complexed with Cas9 protein. There are several advantages of directly delivering CRISPR/Cas9 ribonucleoprotein complexes (RNPs) or in vitro transcripts (IVTs) of gRNA and Cas9 into plant cells. One such advantage is reducing off-target mutations via avoiding insertional mutagenesis or the integration of foreign DNA fragments in the genome. To deliver the RNP complex via microprojectile bombardment, the same concentrations of gRNA and Cas9 are mixed in a 4-5:1 proportion in order to develop the RNP complex and are then subsequently mixed with gold particles, spread onto macrocarriers, and air-dried. This method was successfully used in common wheat for inducing mutations in the desired genes and is expected to function equally well in other crop plants (Liang et al., 2018, 2019; Zhang Y. et al., 2021).

### *Agrobacterium*-Mediated gene delivery method and its modifications

*Agrobacterium*-mediated gene delivery is one of the predominant plant genetic transformation methods. One of the primary reasons for its popularity is the ease with which it can be adopted and implemented in laboratories familiar with plant tissue culture and molecular cloning procedures, without significant resources (Hiei et al., 1997). Bacterial species other than *Agrobacterium*/*Rhizobium* were later identified to be capable of delivering DNA to plant cells (Hooykaas et al., 1977). However, so far, *Agrobacterium* remained the primary vector for DNA transfer.

Two species, *Agrobacterium tumefaciens* and *A. rhizogenes* (*Rhizobium rhizogenes*), and several *A. tumefaciens* strains are used to transform a wide variety of plant species. Collectively, the availability of these strains increased the host range of *Agrobacterium* spp. However, using different *Agrobacterium* strains with different host plants needs lots of optimization, as the co-culture duration and subsequent elimination afterwards is species/genotype-specific. *Agrobacterium tumefaciens* and *R. rhizogenes* also differ in their modes of genetic transformation and use different proteins to mobilize DNA into plant cells (Ream, 2009). *Agrobacterium tumefaciens*-mediated gene delivery yields plants that express transgenes throughout or completely transformed plants (Fernández-Piñán et al., 2019) whereas *R. rhizogenes* produces transgenic hairy roots on wild-type shoots resulting in plants that are a composite of a wild-type shoot with transformed hairy roots (Fernández-Piñán et al., 2019). Therefore, the choice of the *Agrobacterium* species for transformation depends on the experiment’s objective; for instance, *R. rhizogenes* is the vector of choice when the function of the root-specific gene needs to be studied promptly.

In an earlier study, Hooykaas et al. (1977) introduced the *Agrobacterium* Ti plasmid into *Rhizobium trifolii* and found that *R. trifolii* infected Kalanchoe leaves produced tumors, which suggested DNA transfer. Similarly, van Veen et al. transformed *Phyllobacterium myrsinacearum* with the *A. tumefaciens* Ti plasmid and observed that it also induced tumorigenesis in Kalanchoe (van Veen et al., 1988). Later, Broothaerts et al. (2005) demonstrated three other bacteria, *Rhizobium* sp. strain NGR234, *Sinorhizobium meliloti*, and *Mesorhizobium loti* (collectively known as Transbacter^™^) modified with *A. tumefaciens* Ti plasmid to genetically transform *Arabidopsis thaliana*, *Nicotiana tabacum*, and *Oryza sativa* (Broothaerts et al., 2005). However, these bacteria exhibited low transformation efficiencies relative to *A. tumefaciens*, hence they were not used widely in plant transformations. Similarly, but more recently, Rathore and Mullins demonstrated *Ensifer adhaerens* OV14 modified with *A. tumefaciens* Ti plasmid to transform potato, tobacco, *Arabidopsis*, rice, and cassava (for a comprehensive review on this topic, see Rathore and Mullins, 2018). However, *Ensifer* was also shown to be less virulent than *Agrobacterium*. In addition, Cho et al. demonstrated that a phytopathogenic bacteria, *Ochrobactrum haywardense* H1, modified to express the *A. tumefaciens* Ti plasmid, successfully transformed soybean, which otherwise remained challenging to transform using *Agrobacterium* spp. (Cho et al., 2022).

It is apparent from these studies that the so-called “transforming principle,” the Ti/Ri plasmid, was only identified from *Agrobacterium*/*Rhizobium* species and was used to transform different bacterial species to deliver the T-DNA to a wide range of host plants, which was otherwise impossible using *Agrobacterium* due to its specific host range. It will be interesting to see if, in the future, the “transforming principle” will be identified for more bacterial species and if they could be modified similar to *Agrobacterium* to deliver DNA or nucleoprotein complexes to plant, fungal, and animal cells. The common feature among these bacteria is the type IV secretion system that allows delivery of the nucleoprotein complex to the plant cells, followed by its integration in the plant genome. More recently, *Agrobacterium* transformation efficiency and host range was improved by modifying its genome to express *Pseudomonas syringae* type III secretion system (T3SS). Using the engineered *Agrobacterium*, a 250%–400% increase in wheat, alfalfa, and switchgrass transformation is observed (Raman et al., 2022). Moreover, bacteria and fungi that can deliver proteins to plant cells have been identified, and their use as vectors is discussed in the later sections of this review.

### Polyethylene glycol-mediated gene delivery method

Inspired by the successful demonstration of fungal and animal cell transfections via chemical treatment, polyethylene glycol (PEG), poly-L-ornithine, and polyethylenimine have been used in similar experiments performed on plant protoplasts (Altmann et al., 1992; Webb and Morris, 1992; Bilang et al., 1994; Kuwano, Shirataki and Itoh, 2008; Kawai, Hashimoto and Murata, 2010). Polyethylene glycol is a petroleum-derived polyether polymer that exists in various molecular weights. The polymer is hydrophilic and exhibits low biotoxicity; hence, its use for various biological applications. One of its primary uses is as a transfection agent to increase the permeability of the plasma membrane and to improve the transmissibility of charged macromolecules (Altmann et al., 1992). Indeed, PEG in the presence of divalent cations at high pH was demonstrated to deliver naked or liposome-encapsulated DNA into plant protoplasts as early as the 1980s (Altmann et al., 1992). The PEG-mediated delivery of DNA has since been improved significantly (Webb and Morris, 1992; Bilang et al., 1994). Several alterations of the method were tested to improve PEG’s transformation efficiency and frequency and are discussed in the reviews by Altmann et al. (1992), and Webb and Morris, (1992).

There are several advantages associated with the PEG-mediated transformation. This method is technically simple; hence, it allows simultaneous processing of many samples. It utilizes inexpensive supplies and does not have specialized equipment requirements. PEG-mediated transformation is versatile; hence, it does not exhibit the host range limitations of *Agrobacterium*-mediated transformation and could be readily adapted to various plant species and tissue sources with little optimization (Bilang et al., 1994). Additionally, this method is suitable for transient expression of a transgene leading to the production of transgene-free genome-edited mutants. However, the major bottleneck of this method is the regeneration of plant protoplasts into complete plants. Given these advantages, this method has witnessed resurging interest, specifically with the advent of genome-editing procedures, both to test the genome-editing reagents and to regenerate plants with desired mutations after genome editing (Yue et al., 2021). Also, this method has been used to deliver the triplex-forming oligonucleotides (TFOs) or Gene Repair OligoNucleotide (GRON) to induce mutations in plant cells (Gocal, 2014; Gocal et al., 2015; Sauer et al., 2016). Protoplast isolation and transformation with PEG were performed in many crop plants (Bilang et al., 1994) including but not limited to wheat (Brandt et al., 2020), rice (Lin et al., 2020; Bes et al., 2021), maize (Svitashev et al., 2016), potato (Carlsen et al., 2022), and soybean (Patil et al., 2022). Additionally, in recent years, the PEG-mediated protoplast transfection of Cas9 protein and Cas9 complexed with in vitro synthesized guide RNA (ribonucleoprotein, RNP) was successfully used in major row crop and horticultural crops in a quest to establish a DNA-free genome editing platform (Sant'Ana et al., 2020; Subburaj et al., 2022; Liu et al., 2022).

With the rapid and competitive pace of development in the field of plant genome editing, several new breeding technologies have arisen, which aim improved targeting efficiency and lower costs. Among those are the Rapid Trait Development System (RTDS™) by Cibus. The RTDS™ is a transgene-free, precision gene editing platform that utilizes Oligonucleotide-Directed Mutagenesis (ODM) by targeting a specified gene sequence with a Gene Repair Oligonucleotide (GRON). The engineered GRON (typically around 40 bp) shares homology with the target DNA sequence except for one or a few mismatched base pairs (Gocal, 2014; Gocal et al., 2015). The RTDS™ then relies on the plant cell’s endogenous DNA repair machinery to recognize the mismatch and repair the DNA using the GRON as a template (Gocal, 2014). After ODM, the GRON is degraded by the plant cell, reducing the opportunities for off-target mutations. This system is in contrast to the nuclease-based gene editing systems mentioned above, as supplying a nuclease to induce cleavage is not necessary. Furthermore, GRONs do not require a delivery vector, avoiding integration of foreign DNA into the host genome. Similar to gene editing RNPs, however, GRONs can be delivered into protoplasts via PEG-mediated delivery, electroporation (see below) or particle bombardment (see above) (Gocal et al., 2015). According to Gocal et al. (2015), the use of ODM has been demonstrated in *Arabidopsis*, canola, corn, rice, tobacco, and wheat. The simplicity, accuracy, and avoidance of transgene integration renders ODM, and subsequent systems such as the RTDS™, attractive choices for rapid gene editing. Despite these advantages, however, the major bottleneck in implementing this system is a lack of efficient protoplast production, transformation, and regeneration protocols for most crops along with the genotype-dependence of the protoplast regeneration system.

## Unconventional gene delivery methods

Other than the conventionally-used gene delivery methods, there are other methods developed and used by the research community. Some of these methods are elaborated on below.

### Electroporation

Electroporation, a physical, genetic-transformation method, is used to deliver DNA constructs through plasma membranes by producing transient, unstable pores, allowing the transportation of macromolecules such as DNA, RNA, and proteins into cells (Ozyigit, 2020). In plants, protoplasts (Fromm et al., 1985) are often used for electroporation due to the ease of uptake of plasmids for stable or transient genetic transformation. After optimizing electroporation parameters, the method was used successfully to transform embryonic cells (Xu and Li, 1994), zygotes (Li and Yang, 2000), mitochondria (Farre and Araya, 2001), microspores (Brew-Appiah et al., 2013; Bhowmik et al., 2018), and shoot apices (de GarcÃ-a and Villarroel, 2007) in several plant species, such as tobacco, rice, wheat, and maize (for a review, see Barampuram et al., 2011). However, after electroporation-mediated gene delivery, the explant needs to be regenerated to obtain transformed progeny, making the method labor-intensive and genotype-dependent.

More recently, Furuhata et al. demonstrated that proteins (Cre recombinase) could be delivered to cultured *Arabidopsis thaliana* cells with intact cell walls with up to 83% efficiency, which is a step forward for electroporation-mediated plant genetic transformation (Furuhata et al., 2019). In summary, electroporation-mediated plant gene delivery is fast and inexpensive; however, it may need optimization of some parameters, such as field strength, pulse duration, cargo concentration, and explant type to obtain satisfactory transformation efficiencies with minimal damage.

### Magnetofection

Magnetofection has proven to be a simple and efficient method of transforming target animal cells by applying an external magnetic field (Scherer et al., 2002; Plank et al., 2003). The most commonly used magnetofection system comprises superparamagnetic iron oxide nanoparticles coated with the cationic polymer polyethylenimine (PEI), which can bind negatively charged nucleic acid molecules through electrostatic interaction (Zuvin et al., 2019). Magnetic nanoparticle (MNP) mediated delivery can be achieved via associating viral or non-viral vectors with MNPs. The major benefit of magnetofection lies in the rapid and efficient transfection using a relatively low vector dose and the possibility of targeting the vector to a specific explant area under a magnetic field (Plank et al., 2011).

Although magnetofection has been broadly applied in the animal system, only two published articles described the successful application of magnetofection in plants. Specifically, in 2017, Zhao et al. demonstrated the use of magnetofection to introduce plasmid DNA-coated PEI functionalized iron oxide nanoparticles (168 nm in diameter) into pollen grains of cotton, pumpkin, zucchini, *capsicum*, and lily (Zhao et al., 2017). They speculated that the DNA-loaded nanoparticles entered pollens via the apertures (∼5 μm) in the pollen wall. Through pollen magnetofection, both transient transformation and direct production of transgenic seeds without regeneration can be achieved; hence the authors claimed the system is tissue culture- and genotype-independent. However, in 2020, Vejlupkova et al. published an article claiming that the results of the cotton study were irreproducible in monocots, specifically in maize, sorghum, and lily (Vejlupkova et al., 2020). Recently, Wang et al. established a maize pollen transfection system using MNPs for a large-scale, fast, and efficient maize transfection (Wang Z. et al., 2022). Importantly, they pointed out that opening the pollen aperture via pretreatment with the transfection buffer for 10 min and transfection at 8°C (to protect maize pollen viability) is essential for exogenous gene delivery.

Although magnetofection has not yet been accepted as a mainstream genetic transformation method, it possesses some desirable features, such as genotype-independence and low toxicity. If optimized for more crops, it has potential to be used widely.

### Sonication

Sonication-mediated gene delivery employs an ultrasound that can produce a variety of nonthermal bioeffects such as acoustic cavitation and disrupting the cell membrane, permeabilizing it and facilitating the uptake of genetic materials (Joersbo and Brunstedt, 1992; Miller et al., 2002). This technique provides an attractive alternative to other physical methods due to its low cost. It has been reported to allow gene delivery in plant protoplast (Joersbo and Brunstedt, 1990), suspension cells (Liu et al., 2006; Zolghadrnasab et al., 2021), and even intact leaf segments (Zhang et al., 1991). Due to the cavitation, sonication was also combined with other gene delivery methods to reach higher efficiencies, such as sonication-assisted *Agrobacterium*-mediated transformation (SAAT) (Trick and Finer, 1997; Dutta et al., 2013) and sonication-mediated pollen-transformation (Yang et al., 2017). Sonication, being a mechanical method, is less dependent on the explant type. It could be an effective means of delivering DNA to plant cells/tissues, given sonication conditions are optimized to minimize any damage to cells and tissues while providing effective cargo delivery (Liu et al., 2006).

### Silicon carbide whiskers

Silicon carbide (SiC) whisker-mediated transformation in plants was first reported by Kaeppler et al. where small needle-like silicon carbide whiskers are mixed in the liquid medium, usually with a vortex, in the presence of plasmid DNA and target plant cells (Kaeppler et al., 1990; Kaeppler et al., 1992). Transgenic plants such as rice (Matsushita et al., 1999), maize (Frame et al., 1994), and cotton (Asad et al., 2008) were produced using this method. Despite the method being simple, requiring less resources, and being cost-effective, only a handful of papers report stable genetic transformation using this method. SiC whiskers are hazardous to humans (Svensson et al., 1997; Chen et al., 2018); therefore, safer alternatives, such as the aluminum borate whiskers (ABW), were later explored for producing transgenic rice plants (Mizuno et al., 2004). Moreover, ABW was also shown to significantly increase *Agrobacterium* infection efficiency (Nanasato et al., 2011) during the adventitious shoot organogenesis in kabocha squash (*Cucurbita moschata* Duch). Several factors need to be considered when using SiC-mediated transformation: the type of the whiskers, the type of plant cell/tissue, and the potential health hazard the material imposes.

### Use of fungi and bacteria to deliver native or recombinant proteins into plant cells

These methods were initially developed to characterize fungal effector proteins and understand host-pathogen interactions. However, these methods have much potential for delivering other recombinant proteins, such as TAL effector nucleases or zinc-finger nucleases, to edit genomes at the desired site or to transiently express a transcription factor or a regulatory protein. For instance, van der Linde et al. reported a unique “Trojan horse approach” to deliver recombinant proteins to maize using the secretory capabilities of the smut fungus *Ustilago maydis* (van der Linde et al., 2018). This strategy allowed authors to deliver recombinant proteins into individual corn cells at certain cell layers and at a precise time point. The method utilized host-pathogen interactions to transport recombinant proteins to host cells and tissues and, for the first time, demonstrated the potential of filamentous fungi as plant gene delivery vectors.

On the other hand, bacteria with a type III secretion system (T3SS) were used more broadly for direct protein delivery, particularly in mammalian cells for biomedical applications (Ittig et al., 2015; Bai et al., 2018). However, the utility of bacteria with T3SS in plant research is somewhat limited, largely due to the hypersensitive response (HR) induced in the host plants by these vectors. Given this limitation, recombinant strains of HR-inducing and non-HR-inducing pathogens were identified to serve as delivery vehicles. These bacterial vectors are used in eudicots and monocots, and some examples are included here. A variant strain of *Pseudomonas syringae* with a deletion of multiple effectors with reduced hypersensitive response was used in wheat. Also, *Pseudomonas* *fluorescens* with an engineered T3SS and no HR were used to characterize bacterial and fungal effectors in wheat (Yin and Hulbert, 2011). Similarly, Sharma et al. reported using the effector delivery system of the rice pathogen *Burkholderia glumae* to characterize the AVR-Pik and AVR-Pii effectors of the fungal rice pathogen *Magnaporthe oryzae* (Sharma et al., 2013). In line with this study, Upadhyaya et al. demonstrated the use of the wheat pathogen *Xanthomonas translucens* to deliver fusion proteins containing T3SS signals from *P. syringae* (AvrRpm1) and *X. campestris* (AvrBs2) avirulence (Avr) proteins into wheat leaf cells (Upadhyaya et al., 2014). This area of research is evolving, and in due course of time, we may witness more pests/pathogens with similar capabilities will be identified and used in gene product characterization or genome editing.

## Nanoparticle-based gene delivery methods

Several nanoparticle-based technologies have been developed for plant gene delivery in the past 3 years and exhibited many advantages over conventional methods. First, nanoparticles enable plant species-independent delivery of cargoes as the cell entry is hypothesized to be mechanically-driven (Lew et al., 2020a). Second, nanoparticles protect or at least delay the genetic cargo degradation, increasing the active life-time of cargoes in plant tissues and cells, which is especially important for fragile cargoes such as RNA (Shidore et al., 2021). Third, nanoparticle delivery enables the possibility of transient expression without gene insertion into plant host genomes (Demirer et al., 2019a; Wang et al., 2019). This is not only desirable for many research applications where gene function is rapidly studied in planta, but also is a transformative technology for transgene- and GMO-free CRISPR/Cas9 crop engineering (Demirer et al., 2021). Lastly, a wider range of cargo types, including nucleic acids, proteins, small molecules, and agrochemicals, can be delivered with nanoparticles (Ng et al., 2016). There are, however, some limitations of nanoparticles such as lower efficiency, their inability of systemic travel in plant tissues, and limited studies on their environmental safety and accumulation.

Below, we highlight the recent developments in nanoparticle-mediated delivery field covering only the last 3 years. For a comprehensive review of technologies before 2019, readers are encouraged to refer to these cited reviews (Wang et al., 2016; Cunningham et al., 2018; Sanzari, Leone and Ambrosone, 2019; Wang et al., 2021).

**FIGURE 1:**
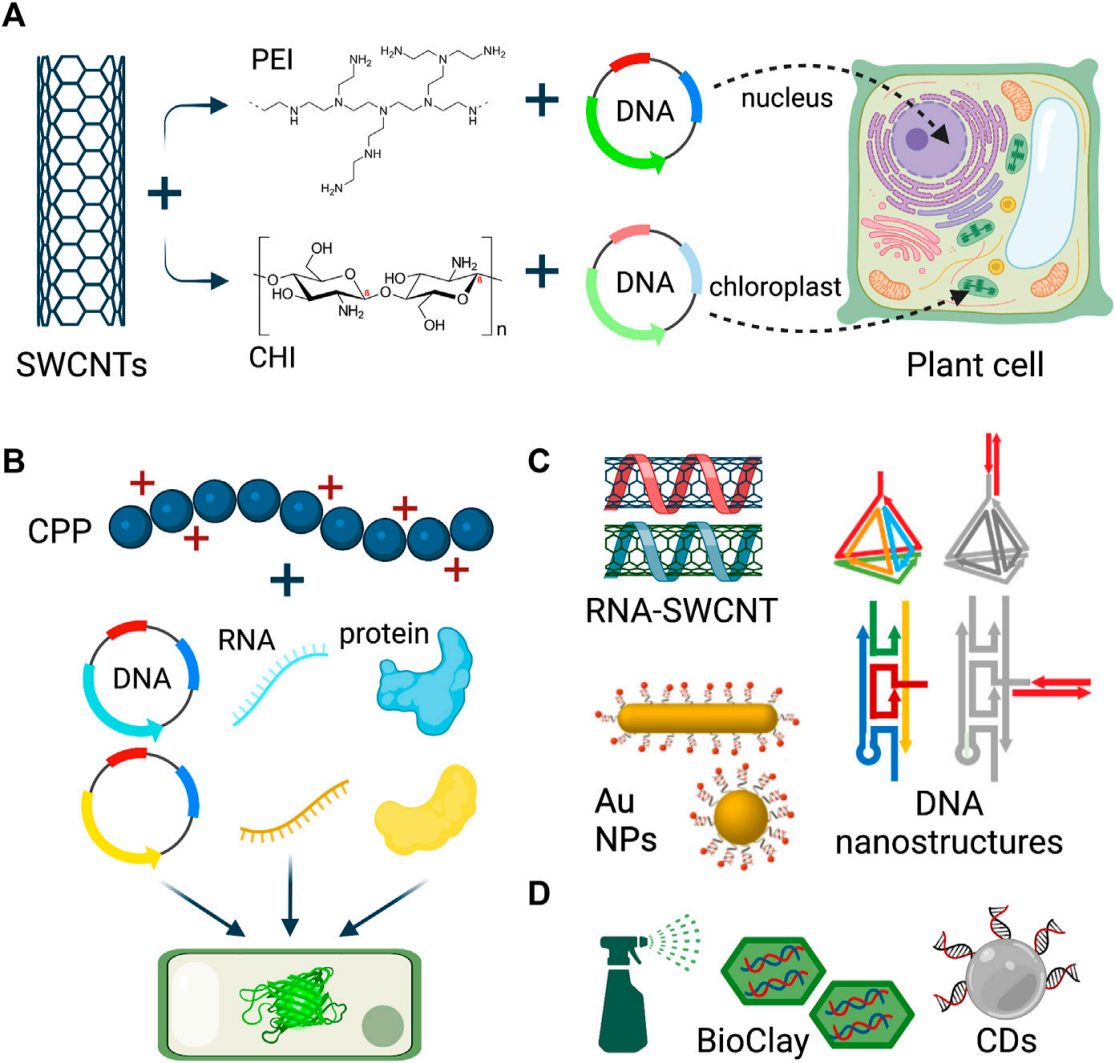
Nanoparticle-mediated delivery to plants. **(A)** Polymer-functionalized single-walled carbon nanotubes (SWCNTs) deliver plasmid DNA into plant nucleus and chloroplast for gene expression. **(B)** Cell penetrating peptides deliver multiple DNA, RNA, and protein cargoes to plant leaves. **(C)** SWCNTs, DNA nanostructures, and gold nanoparticles are used to deliver siRNA and dsRNA to plants via leaf infiltration. **(D)** BioClay and carbon nanodots are topically sprayed on leaves and pollen for dsRNA and siRNA delivery. Figure created with BioRender.com.

One of the well-studied nanomaterial types for plant gene delivery is single-walled carbon nanotubes (SWCNTs). When covalently-functionalized with positively-charged polymers, such as PEI and chitosan (CHI), these nanomaterials were able to deliver electrostatically-attached DNA plasmids into plant nuclei and chloroplasts, respectively (Figure 1A) (Demirer et al., 2019b; Kwak et al., 2019). These first-generation studies with carbon nanomaterials established a transient and plant species-independent delivery in various eudicot and monocot species, in which the reporter gene expression lasted 7–10 days in tobacco, arugula, spinach, wheat, watercress, and cotton. More recently, second-generation studies were carried out to optimize the nanoparticle physical parameters and cargo binding mode to increase the cargo delivery efficiency (Hu et al., 2020; Ali et al., 2022; Sharma and Lew, 2022).

Another hollow nanotube platform called rosette nanotubes (RNTs) were composed via the self-assembly of complementary guanine−cytosine motifs, and these nanoparticles were complexed with plasmid DNA for plant delivery (Cho et al., 2020). RNTs entered wheat microspores and did not affect health, division, or regeneration abilities of microspores. Separate labeling of both the DNA and RNTs showed that while RNTs reached the microspore nucleus, DNA was mostly present in the cell cytosol, which caused low efficiency of gene expression from delivered DNA. However, this study is promising to enable the discovery of nanomaterial formulations that can enter crop microspores.

Numerous nanomaterial platforms have been developed for the delivery of single- and double-stranded RNA cargoes for gene silencing, in addition to the DNA plasmids for gene expression (Figure 1C). For instance, oligo-wrapped SWCNTs were generated to deliver small interfering RNA (siRNA) cargoes to plant leaves to silence endogenous disease susceptibility genes with high efficiency (Demirer et al., 2020). Moreover, DNA nanostructures with programmable size, shape, and stiffness features were established to deliver siRNA into the leaves of tobacco, arugula, and watercress (Zhang et al., 2019a). Carbon and DNA-based nanomaterials are not the only formulations used for plant RNA delivery. Recently, several studies performed siRNA delivery into plant leaves via spherical and rod-shaped gold nanoparticles (Zhang et al., 2022), and with gold nanoclusters (Zhang Y. et al., 2021). It is noteworthy to mention that these studies discovered that nanoparticle cellular internalization was not needed for siRNA-mediated gene silencing and that while rod-shaped gold nanoparticles had higher efficiency of cellular uptake, siRNA delivered via spherical gold nanoparticles induced stronger gene silencing (Zhang et al., 2022).

Nanoparticle-cargo conjugates can be delivered through leaf infiltration using a needless syringe in a lab research context, and for scalable field applications, there are some studies demonstrating the feasibility of topical application of double-stranded RNA (dsRNA) and siRNA cargoes with layered double hydroxide (LDH) nanoparticles (Mitter et al., 2017) and carbon dots (Schwartz et al., 2020) (Figure 1D), which eliminates the issues of the instability of naked RNA sprayed on plants. The LDH platform, which is called BioClay, enabled virus/fungus protection for 21 days when sprayed on virus/fungus-challenged leaves (Mitter et al., 2017; Niño-Sánchez et al., 2022). More recently, the BioClay technology has been topically applied for dsRNA delivery into tomato pollen (Yong et al., 2021), extending the plant tissue types for various applications. In addition to the BioClay, carbon dots (CDs) were used to topically deliver siRNA molecules and generate highly efficient gene silencing in tobacco and tomato leaves (Schwartz et al., 2020).

There has been a substantial amount of progress in the field of nanomaterial-mediated biomolecule delivery to plants in the last three to 4 years. The choice of nanoparticles to use typically depends on the cargo of interest, target plant tissue, and application type. For instance, while SWCNTs are effective in plasmid delivery for transient expression applications in somatic cells, RNTs may be a better option for microspore delivery, and BioClay is highly advantageous for topical RNA delivery. Yet, there is even much more to achieve in the areas of plant CRISPR gene editing using nanoparticle-mediated delivery and stable crop transformations.

## Cell-penetrating peptides

In addition to nanoparticles, cell-penetrating peptides (CPPs), which are short peptides composed of 5–30 amino acids, have shown remarkable abilities to deliver diverse biomolecules, such as DNA, RNA, proteins, and RNP complexes into many plant species (Figure 1B) (Thagun et al., 2020; Zhang S. et al., 2021).

Cationic CPPs are the most commonly used type of peptides, and they can be loaded with negatively charged DNA/nucleic acids through electrostatic interaction and yield transient expression of proteins from DNA cargoes. Similarly, RNA molecules were delivered to plant cells using CPPs, which had increased half-life compared to free RNA. Lastly, CPPs were not only used to deliver nucleic acids, but also proteins and multiple biomolecules to plant cells simultaneously (Zhang S. et al., 2021). Similar to nucleic acids, negatively charged proteins, such as BSA, can be electrostatically grafted onto cationic CPPs. To demonstrate simultaneous multiple cargo delivery, Thagun et al. used a superfolder GFP-based complementation assay with a cytosolic homodimer of the *Coffee arabica* 7-methylxanthine methyltransferase 1 protein. The codelivery of multiple plasmids or proteins with CPPs resulted in the creation of complemented GFP fluorescence in plant leaf cells (Thagun et al., 2020). Similarly, Wang et al. developed a fluorescent complementation-based assay to quantify CPP-mediated protein delivery to plant cells (Wang J. W. et al., 2022).

Compared to nanoparticles, they are more biodegradable, hence potentially have better suitability for field studies. However, most studies are limited to cationic CPPs limiting delivery to only negatively charged cargoes.

## Virus mediated delivery

The use of viruses to deliver DNA to bacterial cells has its roots in the early years of molecular biology. However, the use of viruses to deliver gene expression constructs to eukaryotic cells did not start until much later. The use of disarmed viruses and, more recently, virus-like particles (VLPs) to deliver cargos such as DNA, mRNA, and nucleoprotein complexes is more common in medical research than in plants (for a review, see Zhang S. et al., 2021). However, this method is gaining traction in plant research to determine gene function by expressing/silencing genes, popularly known as virus-induced gene silencing (VIGS) and virus-mediated overexpression (VOX). It is also being used in site-directed mutagenesis using CRISPR-associated nucleases, the technology being dubbed virus-enabled gene editing (VEdGE) or virus-induced genome editing (VIGE) (for a detailed examination of these topics, see Rössner et al., 2022; Gentzel et al., 2022). There are well-established viral gene delivery systems that have been developed and utilized in VIGS, VOX, and VIGE (discussed in Gentzel et al., 2022; Rössner et al., 2022). These include systems based on the cabbage leaf curl virus (CaLCuV, a DNA virus; Baltes et al., 2014), the foxtail mosaic virus (FoMV, a DNA virus; Mei et al., 2019), the broad bean wilt virus2 (BBWV2, a RNA virus; Choi et al., 2019), and a system based on the tobacco rattle virus (TRV, a RNA virus; Khakhar et al., 2021) dubbed ‘VipariNama.’ Emphasis is given to lesser expounded upon viral systems in the following paragraphs.

### DNA virus-based delivery system

Many DNA viruses are used to deliver gene expression cassettes or gene silencing reagents to plants (Peretz et al., 2007; Mei et al., 2019; Rössner et al., 2022), in addition to their application in virus-induced gene silencing. The use of DNA viruses in VIGS is elaborated on in a separate paragraph; also, it has been reviewed recently in Rössner et al. (2022). Therefore, in the following paragraphs, we focused discussion on a unique tomato yellow leaf curl virus (TYLCV)-based delivery system, TraitUP™, to avoid repetition.

Peretz et al. (2007) developed a universal vector system, IL-60, for silencing and exogenous gene expression from TYLCV, a Begomovirus of Geminiviridae. This vector was successfully tested on tomato plants, where it was mechanically injected into the stem. The recombinant virus spreads systemically, but the plants remain asymptomatic. Moreover, the vector did not integrate into the genome, produced no ssDNA progeny, and did not spread to other plants by *B. tabaci*, making it a desirable gene delivery system.

Later, to further improve the cargo capacity of the TYLCV vector system (TraitUP™), the same research group deleted different components of the viral genome and identified a 314-bp intergenic region (IR) as the only viral element required for viral dsDNA replication, and two sense-oriented viral genes (V1 and V2) for its expression and movement (Gover et al., 2014). The authors named this minimal viral construct p1470 and demonstrated its ability to deliver marker genes.

So far, the IL-60 vector system has been successfully used in over 40 plant species belonging to 14 different families including perennial woody plants, such as orange, apple, and grape (Peretz et al., 2007; Cusin, Revers and Maraschin, 2017; Malabarba et al., 2018). The vector was introduced into the plants via root infiltration or by syringe inoculation. The former method was used to express a 6.3 kb bacterial operon that allowed the introduction of a complete metabolic route in tomato plants and the production of an antifungal metabolite, pyrrolnitrine (PRN), rendering them resistant to *Rhizoctonia solani* (Mozes-Koch et al., 2012). This study has demonstrated the ability of this vector system to deliver and express large gene constructs, without a negative impact on its movement and replication, which is an advantage over other viral vectors.

In apple, this technique was successfully used to transiently express the *Malus COP1* gene (Li et al., 2012). It opened an alternative for the functional characterization of apple genes in a homologous system. Cusin et al. (2017) used the IL-60 plasmids in apple for expression of the reporter gene, *GFP*. The ‘Gala’ cultivar of apple treated with pIR-GFP plus p1470 showed a stable, broad, and strong expression of GFP that spread throughout all tissues over time and remained stable for up to 6 months after the plasmid treatment. This early success has motivated the researchers to transfer scab resistance genes (such as *Vf2*), as well as other genes of biotechnological interest, to elite apple cultivars. The genes appeared to be stably expressed throughout the plant and during the course of development. This method eliminated the need for genetic crossing and plant genetic transformation and presented an instant tool for transferring the genetic traits of interest. This gene delivery system has been recently used in grape to study the role of the *VviAGL11* gene in seed morphogenesis (Malabarba et al., 2018). Similarly, in a study conducted in 2018 in Brazil the IL-60 technology was used to introduce an herbicide resistance gene in *Eucalyptus* species.

This technology has opened exciting new possibilities for fast trait delivery in woody fruit species (Nagamangala Kanchiswamy et al., 2015). This episomal expression system, based on modified viruses, may enable the expression of stable genetic traits that can be introduced by treating scions prior to grafting to elite genotypes, bypassing the need for backcrossing to recover the original genetic background.

### RNA virus-based delivery system

To improve the versatility of viral vectors, RNA virus-based delivery methods were developed (Zhang Y. et al., 2019; Ariga, Toki and Ishibashi, 2020; Varanda et al., 2021). Several RNA viruses that were modified to deliver the components of the genome editing machinery (Cas9 and gRNA) to plants are summarized in Table 1. Similar to DNA viruses, RNA viruses were also modified for VIGS. We dealt with it separately in a paragraph; also, the topic was recently reviewed in Rössner et al. (2022).

**TABLE 1:** RNA viruses modified to deliver Cas9 and/or gRNA constructs in plants.

Virus name	Inoculation methods	Virus insert cargo	Host gene target and editing efficiency %	Plants used	Reference
Beet necrotic yellow vein virus (BNYVV)	*Agrobacteria* infiltration	Single gRNA	*NbPDS3*: 85%	Cas9-overexpressing *N. benthamiana*	Jiang et al. (2019)
*Sonchus* yellow net rhabdovirus (SYNV)	*Agrobacteria* infiltration, rub inoculation	Cas9 and single or multiplexed gRNAs	*GFP*: 77–91% *NbPDS*: 40–79% *NbRDR6*: 53–91% *NbSGS3*: 79–91% Multiplexed *NbRDR6* + *NbSGS3*: 60–96%	*N. benthamiana* (WT or GFP expressing)	Ma et al. (2020)
Barley stripe mosaic virus (BSMV)	*Agrobacteria* infiltration; rub inoculation	Single gRNA (+/. FT)	*TaPDS*: 3.8–96.1% *TaGW2*: >75% *TaGASR7*: >70%	*N.benthamiana*; Cas9-expressing wheat	Li et al. (2021)
Potato virus X (PVX)	*Agrobacteria* infiltration; rub inoculation	Cas9 and gRNA	*NbTOM1*	*N. benthamiana*	Ariga, Toki and Ishibashi, (2020)
	*Agrobacteria* infiltration	Single/multiplexed gRNAs+/−tRNA spacers, mobile FT	*NbXT2B*: 37–85% *NbPDS*: 25–73% *NbFT*: 52%	Cas9-expressing *N. benthamiana*	Uranga et al. (2021)
Tobacco mosaic virus (TMV)	*Agrobacteria* infiltration	Individual or simultaneous delivery of Cas9 and single or multiplex gRNAs	*GFP*: 61–63% *NbAGO1*: 6–27% Multiplexed: 11–64%	GFP-expressing *N. benthamiana*	Chiong et al. (2021)
					Cody et al. (2017)
Tobacco rattle virus (TRV)	*Agrobacteria* infiltration	Multiplexed gRNAs with mobile FT or tRNA modifications	*NbPDS3*: 58% *NbAG*: 53–86% Multiplexed: 10–95%	Cas9-expressing *N. benthamiana*	Ellison et al. (2020)
Pea early browning virus (PEBV)	*Agrobacteria* infiltration, rub inoculation	Single or multiple gRNAs	*NbPDS*: 36–72%	Cas9-expressing *N. benthamiana*	Ali et al. (2018)

Jiang et al. (2019) developed a Beet Necrotic Yellow Vein Virus (BNYVV)-based system to deliver gRNA targeting the *PDS* (*Phytoene Desaturase*) gene in Cas9-overexpressing *N*. *benthamiana* plants. It has resulted in 78% photobleaching of the leaf area in the inoculated plants. Similarly, the gene editing capabilities of barley stripe mosaic virus (BSMV) have been demonstrated in *N*. *benthamiana,* wheat, and maize (Hu et al., 2019). After successful delivery of *PDS* gRNA via BSMV to *N*. *benthamiana* plants were co-infiltrated with Cas9 constructs. Hu et al. (2019) further assessed this system with transgenic Cas9-expressing wheat and maize. In wheat, gRNAs targeting the *TaGASR7* gene, determining grain length and weight, exhibited up to 78% mutation efficiency as indicated by restriction digestion of the target gene. Similarly, in maize plants, gRNAs targeting the *ZmTMS5* gene*,* responsible for the heat-induced male-sterile phenotype exhibited up to 48% editing efficiencies (Hu et al., 2019). In a subsequent study, it was shown that multiple BSMV constructs could be co-inoculated to simultaneously target multiple genes in wheat (Li et al., 2021). Further, to address concerns of low gRNA expression by viral vectors, Cody, Scholthof and Mirkov (2017) modified the tobacco mosaic virus (TMV) vector by deleting a coat protein to prevent its systemic movement through the plant and therefore enhanced the local viral titer for transient expression assays.

*GFP* gRNAs when co-infiltrated with the TMV-based Cas9 delivery system showed nearly 70% editing efficiency in GFP-overexpressing *N*. *benthamiana* leaves. In a follow-up study, RNA interference suppressors were used to further optimize the system. In this study, delivering Cas9 and gRNAs from a single TMV construct simultaneously eliminated the need for producing transgenic plants expressing Cas9 or co-delivery of the components (i.e., Cas9 and gRNA) from separate constructs (Chiong, Cody and Scholthof, 2021). Although editing efficiency was lower when using a single construct compared to co-delivery, it was still possible to obtain almost 7% editing efficiency in *N*. *benthamiana* even with a 4.2 kb insert (Chiong, Cody and Scholthof, 2021). In contrast, Ma et al. (2020) reported the highest editing efficiency to date using *Sonchus* Yellow Net Virus (SYNV). Since SYNV can carry a large insert cargo, this characteristic makes it a good candidate for expressing Cas9 as well as single or multiplexed gRNAs. Ma et al. (2020) used this system in *N*. *benthamiana,* where an editing efficiency ranging from 40–91% in plants infected with gRNA constructs targeting *GFP, NbPDS, NbRDR6,* or *NbSGS3* genes was observed. Similar editing efficiency was observed when SYNV constructs designed for the multiplexed editing of the *NbRDR6* and *NbSGS3* genes were used.

Potexviruses (PVX) have also been used as gRNA delivery vehicles. Ariga, Toki and Ishibashi (2020) showed the successful delivery of Cas9 and gRNAs using PVX to *N*. *benthamiana* plants via Agroinfiltration. Furthermore, Cas9 was replaced by this group with a larger base-editing version, which proved to be stably integrated into the virus genome. The PVX vector did not infect the germline or produce edited progeny. However, plants regenerated from mechanically (rub)-inoculated tissues contained *NbTOM1* edits, but with a lower efficiency than those regenerated from Agroinfiltrated-plants (62%). A later study revealed that PVX could be a useful vector to deliver multiplexed gRNAs (Uranga et al., 2021).

In addition, there are two Tobraviruses, TRV and Pea Early-Browning Virus (PEBV), that are currently being used as CRISPR/Cas9 delivery vectors. TRV has a wide host range and has an easily modifiable bipartite positive-sense RNA genome. It is also a proven VIGS vector for several crops. Previous studies had shown that TRV could successfully edit the *PDS3* and *PCNA* genes either singularly or simultaneously in *N*. *benthamiana* when gRNAs were co-delivered from separate constructs (Ali et al., 2015a). Ali et al. (2015a,b) reported that germline *PDS3* editing was observed in seeds collected from the earliest floral buds, obviating plant regeneration from infected tissue. In further testing of this system in Cas9-expressing *Arabidopsis*, TRV delivery of *AtGLI* or *AtTT4* gRNAs produced indels at the target sites (Ali et al., 2018). A direct comparison of TRV versus PEBV editing efficiency of *PDS3* in Cas9-expressing *N*. *benthamiana* revealed that PEBV had a significantly higher editing efficiency of 57–63% compared to 27–35% in the case of TRV (Ali et al., 2018). In recent studies, TRV-delivered gRNAs produced heritable edits when fused with mobile *FLOWERING LOCUS T* (FT) or tRNA sequences targeting *NbPDS* and *NbAG* (Ellison et al., 2020). In another study, TRV gRNA delivery was used to target viral pathogens directly rather than focusing on plant defense-related genes to increase resistance (Ali et al., 2015c). The majority of the research using the TRV-based construct was performed in *N*. *benthamiana*; hence, more research is needed to validate these findings in other crops.

### DNA and RNA virus modifications for virus-induced gene silencing

VIGS is a reverse genetics tool for in vivo gene function studies in plants (Hayward et al., 2011), which depends on post-transcriptional gene silencing (PTGS) machinery in a sequence-specific manner. Briefly, in this process, fragments of a gene of interest are cloned into a virual vector, and the endogenous RNA silencing machinery of the host plant causes RNA degradation and thus reduces the expression of the target gene (Baulcombe, 2004).

VIGS was demonstrated using numerous plant-virus types (Burch-Smith et al., 2004; Kant and Dasgupta, 2019). In the past decade, several viral genomes have been modified to create powerful reverse genetic tools for the functional characterization of genes in plants, such as Tobacco rattle virus (RNA virus, Liu et al., 2002), Apple latent spherical virus (RNA virus, Gedling et al., 2018; Igarashi et al., 2009), African cassava mosaic virus (DNA virus, Lentz et al., 2018), Cucumber mosaic virus (RNA virus, Tzean et al., 2019), barley streak mosaic virus (RNA virus, Bennypaul et al., 2012), to name a few. However, most of the reported VIGS vectors only silence a single gene and VIGS vectors with visible indicators to evaluate early penetrance of the plant tissue are lacking. Soon after its discovery, the VIGS method gained immense popularity and was readily adopted in many plants due to the ease of application and ability to study gene knockout phenotypes (Rössner et al., 2022). Later, some viral vectors were implemented for ectopic gene expression and multigene silencing (Xie et al., 2021), and delivery of guide RNA (Gentzel et al., 2022; Rössner et al., 2022). However, plant studies are lacking in the area of virus-mediated RNA activation (RNAa; Voutila et al., 2012). Transgenerational inheritance of the silencing phenotype was also demonstrated in a few studies (Senthil-Kumar and Mysore, 2011; Bennypaul et al., 2012). Given the large amount of literature available on this topic, which has also been reviewed extensively in several excellent reviews and books (Courdavault and Besseau, 2020; Rössner et al., 2022), only a summary of some commonly used viral VIGS vectors and delivery methods are provided below.

The inoculation step is critical for the successful delivery of the VIGS construct and subsequent steps involving viral proliferation, spread, and gene silencing. The viral construct can be delivered by three different methods: *Agrobacterium*-mediated infiltration, in vivo/in vitro produced RNA infiltration, and DNA infiltration. However, *Agrobacterium*-mediated infection is the most common and convenient method. There are several plant tissues that can be used for *Agrobacterium* infection including sprouts (Yan et al., 2012), roots (Ryu et al., 2004), stems (Wang et al., 2015), leaves (Liu et al., 2002; Liu et al., 2012), the carpopodium of young fruit (Fu et al., 2005), and fruits (Orzaez et al., 2006).

VIGS offers several advantages, including the short period after plant inoculation in which gene silencing effects can be observed. Another advantage of VIGS is that it can be literally applied to any plant species, monocots, and eudicots (Becker and Lange, 2010; Kant and Dasgupta, 2017; Zhang J. et al., 2018; Zhang et al., 2018b), due to the increasing availability of viral vectors. Effective and uniform gene silencing can be achieved when performed at the initial stages of plant development, but the method also works well when applied to induce tissue-specific gene silencing later during development (Stratmann et al., 2011; Bennypaul et al., 2012). VIGS allows the characterization of genes necessary for plant survival that cannot be studied by generating mutants or by stable genetic transformation (Brigneti et al., 2004). With several listed advantages, VIGS also has some limitations. In most cases, VIGS generally does not cause complete silencing of the gene of interest (Sahu et al., 2012; Singh et al., 2015), and the procedure is genotype-dependent. Lastly, the timing of the VIGS phenotype appearance and the duration for which the gene silencing effect lasts is species-specific. Also, an effect of the ambient temperature on VIGS through secondary siRNA production was reported (Fei, Pyott and Molnar, 2021).

## Direct delivery

As mentioned earlier, many methods of gene editing technologies focus on the introduction of raw polynucleotides into a cell. These polynucleotides are then transcribed and/or translated, allowing the native cellular machinery to assemble the gene editing ribonucleoprotein (RNP) complex in vivo. Plasmid expression, however, is not the only approach to introducing RNPs into a cell. Gene editing RNP complexes can be pre-assembled in vitro and then transfected into cells, a process referred to as direct delivery.

Direct delivery of RNPs offers several advantages in gene editing. Most notable perhaps is the lack of dependence on transcription and translation (Andersson et al., 2018; Kim et al., 2018; Liu et al., 2019). RNPs that are delivered directly into cells do not rely on these processes, allowing for editing within cells that have low rates of both (Kuhn et al., 2020; Zhang Y. et al., 2021). Furthermore, independence from transcription and translation allows directly delivered RNPs to show immediate activity after transfection (Kim et al., 2014; Zuris et al., 2015; Kim et al., 2018). In addition to this immediate activity, directly delivered RNPs produce fewer off target effects since they are typically degraded a few hours after transfection, reducing the exposure time and the chances of off target cleavage (Gaj et al., 2012; Liang Z. et al., 2017; Yamagishi et al., 2019; Nicolia et al., 2020; Yang et al., 2021), behaving in a transient manner while still yielding permanent edits. The use of gene-editing RNPs also allows for greater versatility and rapid screening of guide RNAs (Kim et al., 2014; Liang et al., 2015; Tyumentseva et al., 2021), eliminating the need to generate new plasmids for each target loci. Finally, direct delivery of RNPs also avoids integration into nuclear DNA (Kim et al., 2014; Liang Z. et al., 2017; DeWitt, Corn and Carroll, 2017), which can bypass regulatory hurdles and alleviate public concern over transgenic organisms (Woo et al., 2015).

There are limitations, however, to the RNP direct delivery method. For example, not all gene-editing RNPs function equally well due to unknown reasons when they are not expressed from plasmids (Vakulskas et al., 2018). Low cell viability has also been reported as a complication of direct delivery (Yamagishi et al., 2019; Tyumentseva et al., 2021). Direct delivery of RNPs, may also in some cases, require repeated transfections and large amounts of RNPs to be effective (Liang et al., 2015; Tyumentseva et al., 2021). Despite these challenges, direct delivery of RNPs holds great promise for quick, versatile, and accurate genome editing. Several crops have been modified utilizing direct delivery of various RNPs (Table 2).

**TABLE 2:** Summary of various studies concerning the direct delivery of RNPs.

Crop	Explant	Target gene	RNP	Method of transfection	Mutation frequency (respectively)	Reference
Apple (*Malus domestica*) ‘Golden Delicious’	Protoplasts	*MdDIPM-1, MdDIPM-2, MdDIPM-4*	Cas9	PEG 4000	6.70%, 3.30%, 6.10% at 3:1 ratio	Malnoy et al. (2016)
*Arabidopsis thaliana* Ecotype Columbia-0	Protoplasts	*AtPHYB*, *AtBRI1*	Cas9	PEG	16%, *N/A*	Woo et al. (2015)
Cabbage (*Brassica oleracea* var. *capitate*) ‘Dongbok’	Protoplasts	*BoPDS1*	Cas9	Neon electroporation, PEG	3.40%, 1.80%	Lee et al. (2020)
Cabbage (*Brassica oleracea* var. *capitata*) ‘Dae Bak Na’	Protoplasts	*BoGI*	Cas9	PEG	2%	Park et al. (2019)
White cabbage (*Brassica oleracea* var. *Capitata* f. *alba*) ‘Varaždinsko’	Protoplasts	*BoFRI*, *BoPDS*	Cas9	PEG 4000	0.09–2.25%	Murovec et al. (2018)
Chinese cabbage (*Brassica rapa* subsp. *Pekinensis*)	Protoplasts	*BrFRI*, *BrPDS*	Cas9	PEG 4000	1.15–24.51%	Murovec et al. (2018)
Grape (*Vitis vinifera*) ‘Chardonnay’	Protoplasts	*VvMLO-7*	Cas9	PEG 4000	0.10%	Malnoy et al. (2016)
Lettuce (*Lactuca sativa*) ‘Cheongchima’	Protoplasts	*LsBIN2*	Cas9	PEG	46%	Woo et al. (2015)
Maize (*Zea mays*) ‘Hi-II’	Embryos	*ZmLIG*, *ZmALS2*, *ZmMS26*, *ZmMS45*	Cas9 + *ODP2*, *WUS*, & *MOPAT-DSRED* fusion	Particle bombardment	0.57%, 0.45%, 0.21%, 0.69%	Svitashev et al. (2016)
*Petunia* x *hybrida* ‘Madness’	Protoplasts	*PhNR* locus sites 1-6	Cas9	PEG	14%, 19%, 0%, 2.40%, 0%, 20%	Subburaj et al. (2016)
Potato (*Solanum tuberosum*) ‘Desiree’	Protoplasts	*StPPO2*	Cas9	PEG 4000	27% and 68%	González et al. (2020)
Potato (*Solanum tuberosum*) ‘Kuras’	Protoplasts	*StGBSS*	Cas9	PEG, PEG 4000	9% *via* cr-RNP, 25% *via* IVT-RNP	Andersson et al. (2018)
Rapeseed (*Brassica napus*) ‘Topaz’	Protoplasts	*BnFRI*, *BnPDS*	Cas9	PEG 4000	0%	Murovec et al. (2018)
Rice (*Oryza sativa*) ‘Dongjin’	Protoplasts	*OsP450*, *OsDWD1*	Cas9	PEG	19%, 8.40%	Woo et al. (2015)
Rice (*Oryza sativa*) ‘Nipponbare’ & ‘Yukihikari’	Zygotes	*OsDL*, *OsGW7*, *OsGCS1*	Cas9	PEG–Ca^2+^	∼14%, 21.40%, 64.30%	Toda et al. (2019)
Soybean (*Glycine max*) ‘William’	Protoplasts	*GmFAD2-1A, GmFAD2-1B*	LbCas12a, AsCas12a	PEG	0.00–11.70%, 9.10% *via* LbCas12a 0.00–1.60%, 0.60% *via* AsCas12a	Kim et al. (2017)
Tobacco (*Nicotiana attenuata*)	Protoplasts	*NaAOC*	LbCas12a, AsCas12a	PEG	*N/A*	Kim et al. (2017)
Tobacco (*Nicotiana attenuata*)	Protoplasts	*NaAOC*	Cas9	PEG	44%	Woo et al. (2015)
Tobacco (*Nicotiana benthamiana*)	Protoplasts	*NbALS2*	TALENs ALS2T1L & ALS2T1R	PEG	1.40%	Luo et al. (2015)
Tobacco (*Nicotiana tabacum*) <transgenic>	Protoplasts	Transgenic I-SecI site	I-SceI, I-SceI + Trex2-DNA	PEG	0.00%, 7.70%	Luo et al. (2015)
Tomato (*Solanum lycopersicum* L.)	Protoplasts	*SlCCD7*, *SlCCD8*	Cas9	PEG	30%, 90%	Nicolia et al. (2020)
Wheat (*Triticum aestivum* L.)	Microspores, embryos	*TaIPK1*	ZFN	Cell-penetrating peptides	See reference	Bilichak et al. (2019)
Wheat (*Triticum aestivum* L.) ‘Kenong 199’	Protoplasts	*TaGW2-A1, TaGW2-B1, TaGW2-D1*, *TaGASR7*	Cas9	PEG	5.70%, 33.40%, 21.80%, 45.30%	Liang et al. (2017a)
Wheat (*Triticum aestivum* L.) ‘Kenong 199’	Immature embryos	*TaGW2-A1, TaGW2-B1, TaGW2-D1*, *TaGASR7*	Cas9	Particle bombardment	0.03%, 0.18%, 0.21%, 0.56%	Liang et al. (2017a)
Wheat (*Triticum aestivum* L.) ‘YZ814’	Immature embryos	*TaGW2*, *TaGASR7*	Cas9	Particle bombardment	1.30%, 1.80%	Liang et al. (2017a)

Direct delivery is not only employed in gene editing but can also be used for gene regulation. For example, double-stranded RNA (dsRNA) can be directly delivered to cells via exogenous application to induce RNA interference (RNAi) (Dubrovina and Kiselev, 2019; Das and Sherif, 2020). Unlike nuclease-based and ODM-based systems (see above), exogenously applied dsRNA and subsequent RNAi do not act on the plant’s genome, but rather on the plant’s transcriptome. This strategy avoids several disadvantages of gene editing including transgene integration (Fletcher et al., 2020), the dependency on nuclear delivery for functionality (Dudley and Goldstein, 2003; Glass et al., 2018), and explant regeneration (Tenllado, 2004; Dubrovina and Kiselev, 2019). With these advantages, RNAi *via* directly delivered dsRNA is an attractive option for studying gene function and for regulating gene expression without altering the genome.

Given the ease of application and the effectiveness of the technology, it is only natural that RNAi *via* directly delivered dsRNA be examined for commercialization and practical field applications such as biopesticides. This approach, referred to as spray-induced gene silencing (SIGS) (Wang and Jin, 2017; Christiaens et al., 2020) utilizes the direct delivery of dsRNA for non-transgenic gene silencing in target pests and pathogens. The first such sprayable, dsRNA-based biopesticide developed was Calantha™ by Greenlight Biosciences. The active ingredient in Calantha™ is Ledprona, a 490 bp dsRNA designed to specifically target the Colorado potato beetle (*Leptinotarsa decemlineata*) (Rodrigues et al., 2021). This novel, foliar-applied biopesticide represents a new mode-of-action class of pesticides. Bayer’s (formerly Monsanto) BioDirect™ also represents a SIGS-based bioproduct, targeting *Varroa destructor* mites for the protection of honeybees (Guan et al., 2021; De Schutter et al., 2022).

While this technology holds promise for innovative and more environmentally friendly pest management compared to using chemical pesticides, there are a few limitations that must be addressed before directly delivered dsRNA bioproducts become commercially widespread. Perhaps most importantly, the dsRNAs must withstand environmental degradation long enough to be effective (Mezzetti et al., 2020). Coating of dsRNA onto the layered double hydroxide (LDH) clay nanosheets, dubbed BioClay, offers a solution as it slows down dsRNA degradation (Mitter et al., 2017). Another challenge is that different plant organs vary in their absorption capacity of exogenously applied dsRNAs, possibly hindering RNAi (Das and Sherif, 2020). Potential off-target effects also raise biosafety concerns (Senthil-Kumar and Mysore, 2011). Lastly, insect ingestion of dsRNAs varies by species, complicating broad-scale application (Christiaens et al., 2020). Despite these challenges, direct delivery of dsRNA remains a promising new frontier in gene regulation.

Similar to dsRNAs, Fluoroarabino Nucleic Acid Antisense Oligonucleotides (FANA ASOs) offer the opportunity for gene regulation *via* direct delivery without implementing gene editing. FANA ASOs are single-stranded nucleic acids designed to bind to a specified RNA by complementary base pairing. The FANA ASO/RNA complex is then recognized and cleaved by RNase H, leaving the fragmented mRNA to be further degraded by the cell’s nucleases (Kalota, 2006; Sandoval-Mojica et al., 2021). Unlike dsRNA-induced-RNAi which relies on Dicer and RISC, FANA ASOs rely on RNase H (native to both prokaryotes and eukaryotes), simplifying a two-step reaction into a one-step reaction and expanding the host-range (Sandoval-Mojica et al., 2021). FANA ASOs have also proven to be more stable than dsRNAs (Ferrari et al., 2006; Hunter et al., 2021) and have shown activity in both the cytoplasm and the nucleus (Liang X.-H. et al., 2017). Perhaps the most intriguing (and most useful) property of FANA ASOs is their capacity for self-delivery (gymnosis), independent of transfection agents (Soifer et al., 2011; Souleimanian et al., 2012; Pelisch et al., 2021). These properties have spurred great interest in utilizing FANA ASOs in a wide array of fields and are of particular interest in biopesticide applications (Hunter et al., 2021; Sandoval-Mojica et al., 2021), especially given the ability of FANA ASOs to target bacteria. Commercialization of the FANA ASO technology for research purposes is underway, as seen in AUM BioTech’s extensive product catalog of over 300,000 customized RNA silencing products (www.businesswire.com, 2018). Products designed for use by the average consumer will certainly follow.

In summary, the direct delivery of ribonucleoproteins, dsRNA, and FANA antisense oligos is a feasible method for inducing DNA-free, transgene-free gene editing and gene regulation. While mutation frequencies may not be high across species and transfection methods, direct delivery will allow researchers to avoid many of the regulatory hurdles that currently plague today’s biotechnological legal landscape. Transgene-free products are also more favored by consumers, which will hopefully bolster the public’s support of this continuing research and development.

## Pollen/microspore-mediated gene delivery

A wide range of pollen or immature pollen (also known as microspores–single-celled) transformation procedures were developed and tested in a variety of plant species to use pollen/microspores as a “super vector” for transgene delivery (Resch and Touraev, 2011). However, whether the foreign DNA integrates into the genome within pollens/microspores or remains as an episome and later gets integrated into the egg or central cell genome after double fertilization is still controversial and needs further research (Resch and Touraev, 2011). Irrespective of the timing of transgene integration in the genome, the use of pollen as a vector for transgene delivery has several advantages, such as immature pollens are unicellular and haploid and hence could be used to regenerate doubled haploid (homozygous) plants via tissue culture or could mature into pollens and used to artificially pollinate plants. Microspores/pollens exist as a synchronous mass of a single/defined number of haploid cells in large numbers that could be transformed in various ways to deliver transgenes to desired plants. In the past, mature pollens (two or three celled structures) were transformed in various ways such as incubation in DNA solutions, biolistics, co-cultivation with *Agrobacterium*, electroporation, and liposome-mediated delivery (for a review, see Resch and Touraev, 2011). These approaches were also attempted on microspores. For instance, a biolistic method called “Male germline transformation (MAGELITR)” was developed, where the unicellular microspores were bombarded with microprojectiles coated with DNA and transfected microspores were matured into pollen grains for use in artificial pollination. Transgenic plants were obtained by germinating the seeds from artificially pollinated plants on a selection medium (Touraev et al., 1997). Similarly, wheat, barley, and corn microspores were electroporated, co-cultured with *Agrobacterium*, or exposed to PEG and later induced to produce pollen embryoids and mature transgenic plants (c.f., Brew-Appiah et al., 2013; Rustgi et al., 2017, 2020; Kumlehn et al., 2006; Folling and Olesen, 2001; Bhowmik et al., 2018).

More recently, pollens were transformed using different nanoparticle carriers, such as rosette nanotubes (RNTs) in wheat (Cho et al., 2020), sheet-like clay nanoparticles in tomato (Yong et al., 2021), single-walled carbon nanotubes (SWNTs) in oil palm (Lew et al., 2020b), cationic cell-penetrating peptides (CPP) in wheat (Bilichak, Luu and Eudes, 2015) and triticale (Chugh, Amundsen and Eudes, 2009; Pepper et al., 2017), and iron oxide magnetic nanoparticles (MNPs) in cotton (Zhao et al., 2017) and maize (Wang et al., 2022). However, like many other pollen transformation methods, some of these methods remain controversial due to the inability to reproduce results or obtain similar results in a different plant system or by a different research group. Hence, more work is needed to enable routine and widespread use of these methods.

The HI-Edit method is another unique pollen-mediated gene delivery method, where genome-editing reagents are delivered through a haploid inducer line to a conventional genotype (Kelliher et al., 2019). This process allows delivery of the genome-editing reagents to the egg cell, followed by the loss of the paternal genome. The genome-editing reagents induce mutations at the target site in the haploid maternal genome, which is later doubled using antimitotic drugs. This procedure allows the development of homozygous mutant lines in wheat in a single generation (Kelliher et al., 2019). The applicability of this unique system in other plant species still needs to be evaluated.

## Perspective

Biomacromolecule delivery methods to plants were and are an area of significant interest. With the advent of relatively user-friendly genome editing methods, the delivery of these reagents has further reinvigorated the interests in developing and testing different delivery methods. The demands from a cargo delivery method have also changed over time from the requirement of stable transformations (genomic integrations) to transient expression, and thus from strictly DNA delivery to delivery of DNA/RNA, protein, or nucleoprotein complexes. As mentioned in this review, a substantial progress has been made over the last four decades. However, there is still scope for improvements to cover the specific future needs to transiently express CRISPR-associated proteins to allow editing of genes in reproductive or meristematic plant tissues to be inherited in progeny (seed/clonally propagated) and transiently expressed in a spatiotemporally-controlled manner in certain circumstances or tissues. This will allow for studying the gene’s function or generating tolerance to biotic/abiotic stresses (CRISPR-based tissue-specific knockout system, CRISPR-TSKO; Decaestecker et al., 2019). This area of transient delivery of DNA, RNA (mRNA, siRNA, miRNA, dsRNA), protein/peptide has vast research opportunities. Other than the conventional single gene or a few gene edits or introductions, the science of genetic engineering is steering in the direction of a systems approach, i.e., engineering whole new pathways or modifying the existing ones to serve a purpose. This need has also created a demand to look for engineering options at a different scale and deliver the macromolecular drivers of such changes to plant cells. The following two paragraphs talk about such needs.

Besides linear DNA, there are other biomorphic DNA, such as circular DNA that can be employed for genetic engineering in cells. There is substantial evidence in the literature to support the existence and maintenance of extrachromosomal circular DNA (eccDNA, collectively known as mobilome/circulome) in the nucleus and mitochondria of plant and animal cells (Zuo et al., 2022). These eccDNAs could be engineered to express a transgene or used to install a battery of transgenes to achieve biomimetic engineering. Additionally, the artificially-engineered plant mini-chromosomes or recombinant naturally existing extranumerary/supernumerary/B-chromosomes could be used to install new pathways to improve the fundamental plant processes of photosynthesis and nitrogen fixation, or to produce biodiesel and biomaterials (Birchler et al., 2016). The delivery of these enormous macromolecular complexes could open up new avenues of exciting research.

The transplantation of the nucleus after making desired alterations into the desired cytoplasm or a hybrid nucleus in the cytoplasm of a third genotype (tri-parental fusion) to test a gene’s effect in different maternal environments has been attempted in the past. This could be an area of interest to widen our understanding of the effect of maternal inheritance on the expression of nuclear genes (retrograde signaling). This gene delivery method has been briefly used in the past (Saxena et al., 1986), but might be highly relevant in the future with new synthetic biology tools. Also, the possibility of producing progeny of two males and a female parent was demonstrated in maize (Grossniklaus, 2017; Dresselhaus and Johnson, 2018). It is a route that could be taken to deliver gene editing reagents to a plant genome to make desired genomic alterations. More recently, Cournoyer et al. (2022) engineered artificial photosynthetic yeast cells by introducing cyanobacterial cells in yeast protoplast (spheroplast) to study the evolution of photosynthetic eukaryotic cells. We anticipate this novel gene delivery method could be used to further our understanding of retrograde signaling between the organellar and nuclear genomes in plants and to one-day engineer new lifeforms to colonize space. In conclusion, despite some progress in the last couple of years in delivering macromolecules to organellar genomes using nanoparticles (Savage, 2022), the delivery methods to organellar genomes are limited, but it is a field that is expected to evolve in the future due to the implications of producing transplastomic plants in some cases over transgenic plants. Lastly, tri-parental inheritance combined with transplastomics will have even more significant implications for future research.
